# Real-ECG extraction and stroke volume from MR-Compatible 12-lead ECGs; testing during stress, in PVC and in AF patients

**DOI:** 10.1186/1532-429X-13-S1-P6

**Published:** 2011-02-02

**Authors:** Zion Tsz Ho Tse, Charles L Dumoulin, Gari Clifford, Michael Jerosch-Herold, Daniel Kacher, Raymond Kwong, William Gregory Stevenson, Ehud Jeruham Schmidt

**Affiliations:** 1 Brigham and Women's Hospital, Boston, MA, USA; 2 University of Cincinnati College of Medicine, Cincinnati, OH, USA; 3 University of Oxford, Oxford, UK

## Background

Due to the Magneto-Hydro-Dynamic (MHD) effect, blood flow within the MRI’s magnetic field (B_0_) produces a large voltage during the S-T cardiac segment [1]. The peak MHD voltage (V_MHD_) can be comparable, in higher-field MRIs, to the R-wave amplitude of the real Electrocardiogram (ECG_real_), so that V_MHD_ reduces ECG-gating reliability and prevents ischemia-monitoring during cardiac imaging/interventions. We hypothesized that (1) separation of ECG_real_ and V_MHD_ from 12-lead ECGs acquired within a 1.5T MRI could be achieved, using adaptive filtering, based on a set of ECG calibration measurements, and (2) a non-invasive beat-to-beat stroke-volume estimation could be achieved from time-integrated systolic V_MHD_.

## Methods

**Figure 1:**
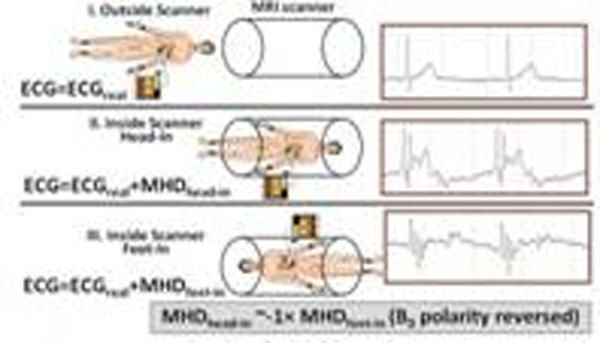
ECGs measured at 3 positions; outside the field (i) and at isocenter with head-in (ii) and feet-in (iii).

**Figure 2:**
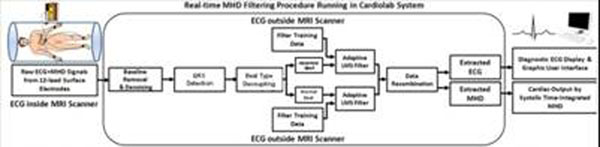
Adaptive filtering diagram used for intermittent PVC patients, with beats separated and then processed independently at abnormal/normal beat filters.

Fig.1 shows 3 sets of 20-sec breath-held ECGs measured at positions (i), (ii) and (iii), utilizing an MRI-compatible Cardiolab-IT digital ECG-recording system [2]. The adaptive filtering procedure was tested in 5 healthy subjects, and 2 patients with Premature Ventricle Contractions (PVCs) and Atrial Fibrillation (AF). Validation was based on comparing the filter-derived ECG_real_ with ECGs measured periodically outside the MRI. The data processing block diagram (Fig. 2) includes training of adaptive Least-Mean-Square filters with ECG_real_ input (i), application of the trained filters to ECGs acquired in (ii) and (iii), which separates the V_MHD_ from ECG_real_.

## Results

**Figure 3:**
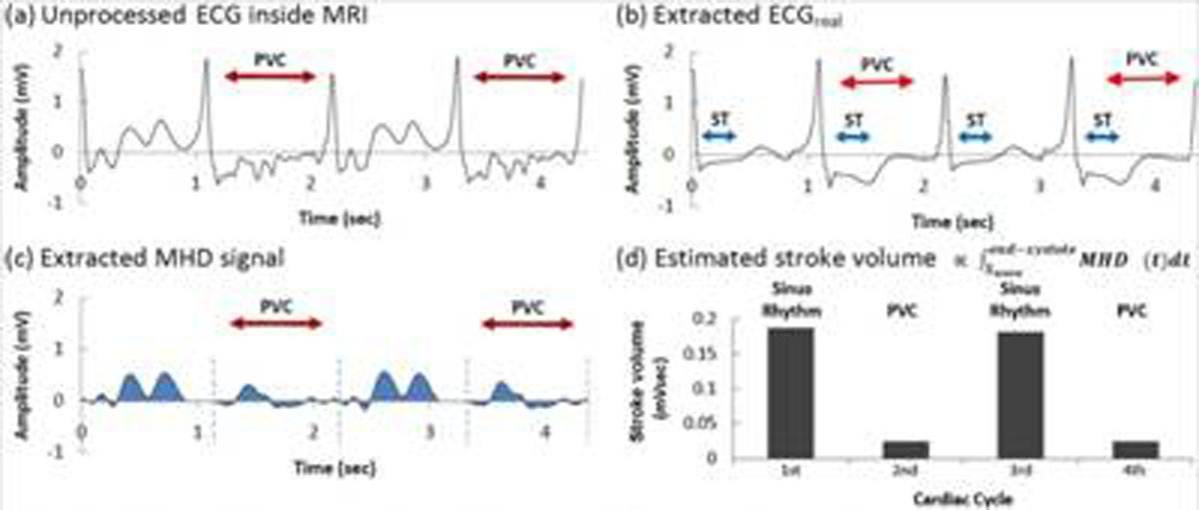
Results from a PVC patient (Ejection Fraction 20-25%, mitral regurgitation.

**Figure 4:**
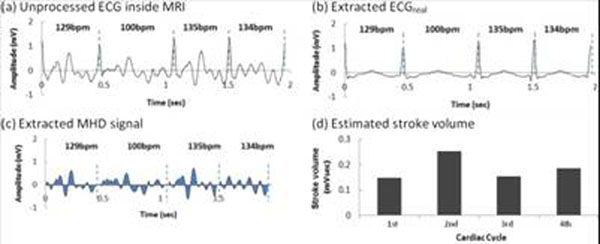
Results from AF patient (100-150 bpm)

**Figure 5:**
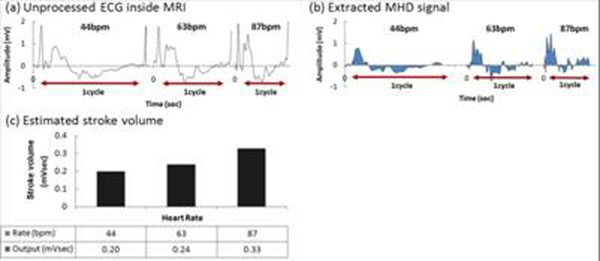
Results from athlete subject during treadmill stress test (44-87 bpm)

**Figure 6:**
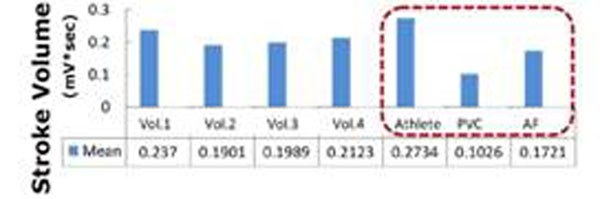
Stroke-volume comparison (cardiac cycles n=20 per subject). Athlete (+24%), PVC (-54%) and AF (-23%), relative to average of volunteers.

PVC patient’s results (Fig. 3): (a) unprocessed surface-lead V6, (b) extracted ECG_real_, and (c) V_MHD_. In (b) S-T segment voltage is restored, and the R-wave dominates for gating. Aortic-flow vortices (c) generate oscillating-polarity V_MHD_, with V_MHD_ peaking during S-T segment. Cardiac beat-to-beat stroke volume (d) was estimated from time-integrated systolic V_MHD_. PVC beats produce substantially lower stroke volume than during sinus-rhythm. AF patient results (Fig. 4): (c) Irregular V_MHD_ and (d) irregular stroke volume are due to ventricular-filling differences at varying heart rates (100-140bpm). Athlete subject results (Fig. 5): Filter tracking of rapid heart-rate changes from 44bpm to 87bpm is shown during a treadmill stress test performed inside the MRI. V_MHD_ (b) and stroke volume (c) increase with heart rate, suggesting that the cardiac output matches higher demand. A stroke-volume comparison of all subjects (Fig 6), derived from time-integrated systolic V_MHD_s, demonstrates the measurement’s sensitivity to pathology.

## Conclusions

The filtering extracts ECG_real_ from measured 12-lead ECG, preserving ECG_real_ for ischemia monitoring and MRI gating. Stroke volume can be non-invasively derived from the time-integrated systolic V_MHD_.
